# Guanidine-HCl Dependent Structural Unfolding of M-Crystallin: Fluctuating Native State Like Topologies and Intermolecular Association

**DOI:** 10.1371/journal.pone.0042948

**Published:** 2012-12-17

**Authors:** Ravi Pratap Barnwal, Geetika Agarwal, Kandala V. R. Chary

**Affiliations:** Department of Chemical Sciences, Tata Institute of Fundamental Research, Colaba, Mumbai, India; Griffith University, Australia

## Abstract

Numerous experimental techniques and computational studies, proposed in recent times, have revolutionized the understanding of protein-folding paradigm. The complete understanding of protein folding and intermediates are of medical relevance, as the aggregation of misfolding proteins underlies various diseases, including some neurodegenerative disorders. Here, we describe the unfolding of M-crystallin, a βγ-crystallin homologue protein from archaea, from its native state to its denatured state using multidimensional NMR and other biophysical techniques. The protein, which was earlier characterized to be a predominantly β-sheet protein in its native state, shows different structural propensities (α and β), under different denaturing conditions. In 2 M GdmCl, the protein starts showing two distinct sets of peaks, with one arising from a partially unfolded state and the other from a completely folded state. The native secondary structural elements start disappearing as the denaturant concentration approaches 4 M. Subsequently, the protein is completely unfolded when the denaturant concentration is 6 M. The ^15^N relaxation data (T_1_/T_2_), heteronuclear ^1^H-^15^N Overhauser effects (nOes), NOESY data, and other biophysical data taken together indicate that the protein shows a consistent, gradual change in its structural and motional preferences with increasing GdmCl concentration.

## Introduction

The phenomenon of protein folding-unfolding and its relevance to diseases [1], [2], [3], [4], [5], [6] is yet to be understood. Protein folding always starts enthalpically/entropically from a state, which is non-native or denatured state and goes into a stable state i.e., native state. Deciphering the structural and dynamic basis of how a given protein sequence translates into a folded conformation requires understanding of the intermediate state(s) of the protein during the process of protein folding/unfolding. The information about the structure and dynamics of different protein states is useful to understand the enthalpically or entropically driven folding/unfolding processes in a given protein, which in turn help in the understanding of the design of protein function. In this endeavor, solution structures are ideally suited for studying such functional states. Earlier studies suggest that proteins exhibit different secondary structural elements under extreme conditions, and sometimes even possesses residual structures [2], [7], [8], [9], [10], [11], [12], [13].

On the other hand, the eye-lens, its architecture and its evolution has been subjects of research for quite a long time [14], [15]. The transparency of the lens is due to its complex architecture and due to the presence of high protein concentration. The function of the eye-lens is to focus the incoming light on the retina. The lens proteins, namely crystallins, accomplish this task of focusing the incoming light. Eye-lens crystallins are of three kinds; α-, β- and γ- crystallins. The lens α-crystallins are known to act as molecular chaperones, while lens β- and γ-crystallins, which were thought to be structural proteins, were shown recently to have diverse roles in various cellular processes [16], [17]. The β- and γ-crystallins are grouped under βγ-crystallin superfamily, because of their structural similarity and these proteins comprise of two consecutive Greek key motifs, which together form a stable eight-stranded β-sheeted sandwich structure [18], [19]. Some of the βγ-crystallins that include several bacterial homologues have been shown to bind Ca^2+^ [20], [21], [22], [23], [24]. When the eye-lens becomes opaque, it results in cataract, a disorder of the eye. Causes of cataract include: mutation in one of the lens-crystallin proteins, ageing process, diabetes, and environmental factors (i.e., change in the pH, temperature and UV-exposure). During these processes, unfolding of any crystallin protein may occur and lead to protein aggregation or altered interaction/association between native crystallins, due to insolubility of proteins. Therefore, it is important to study the complete unfolding pathway of these proteins, which could aid in understanding cataract related problems.

In this backdrop, we have used M-crystallin, a putative βγ-crystallin protein (accession NP_617429) from the genome of an archaea *Methanosarcina acetivorans*, as a model protein to study the folding/unfolding behavior of this oldest relative of βγ-crystallins (Figure S1) [25]. We have earlier solved the 3D structure of M-crystallin in its Ca^2+^–bound form by NMR [25]. A temperature dependent oligomerization of this protein has been reported to have significance in the cataract [26]. The ^15^N relaxation data (T_1_/T_2_), heteronuclear ^1^H-^15^N Overhauser effects (nOes), NOESY data, and other biophysical data of M-crystallin taken together indicate that the protein shows a consistent, gradual change in its structural and motional preferences with increasing GdmCl concentration.

## Results and Discussion

### Resonance assignments of M-crystallin in 4 and 6 M GdmCl

**Figure 1:**
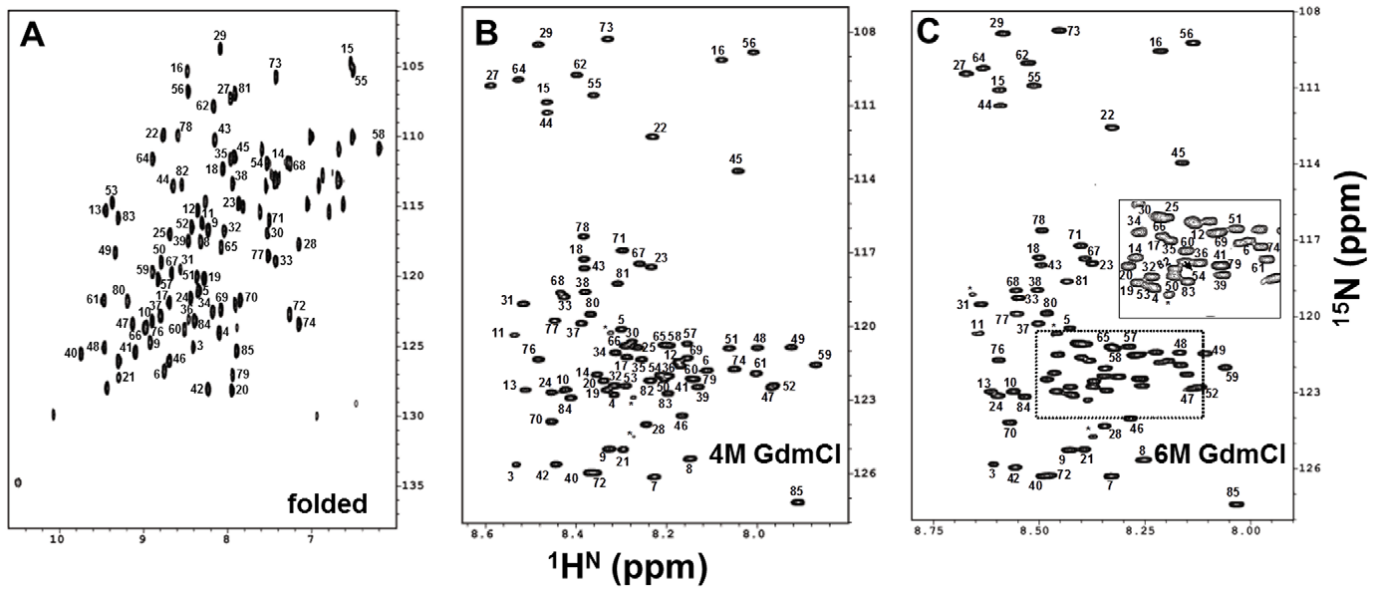
Sensitivity-enhanced 2D [^15^N-^1^H]-HSQC of M-crystallin in the presence of (A) calcium at pH 7.5 and 298 K (folded), (B) 4 M GdmCl, and (C) 6 M GdmCl, at pH 5.5 and 298 K. These spectra were recorded on Bruker Avance 800 MHz spectrometer with 128 and 1024 points along t_1_ and t_2_, dimensions, respectively. Individual peak assignments are shown by the corresponding single-letter code of the amino acid residue and its sequence number along the primary sequence.

The backbone ^1^H, ^13^C and ^15^N resonance assignments of M-crystallin in 4 and 6 M GdmCl were carried out to an extent of 94 and 96%, respectively, using a suite of 3D experiments (HNCACB, HN(CO)CACB, HNCO and HN(CA)CO), as reported earlier [27], [28]. Inherent spectral overlaps caused by the chemical shift degeneracies in ^1^H and ^13^C spins could be resolved using other experiments [29], [30], [31], namely (3, 2)D HNHA, (3, 2)D HNHB, (3, 2)D CB(CACO)NHN, (3, 2)D CT-HCCH-COSY (Figure 1 and Figures S2, S3A/B). All the chemical shifts, thus obtained, were deposited in BMRB under the accession numbers 15918 and 15934, respectively. The spectral signatures of the first two N-terminal residues (M1 and A2), were absent under both the denaturing conditions, as in the native state.

### GdmCl induced unfolding as studied by CD and fluorescence

**Figure 2:**
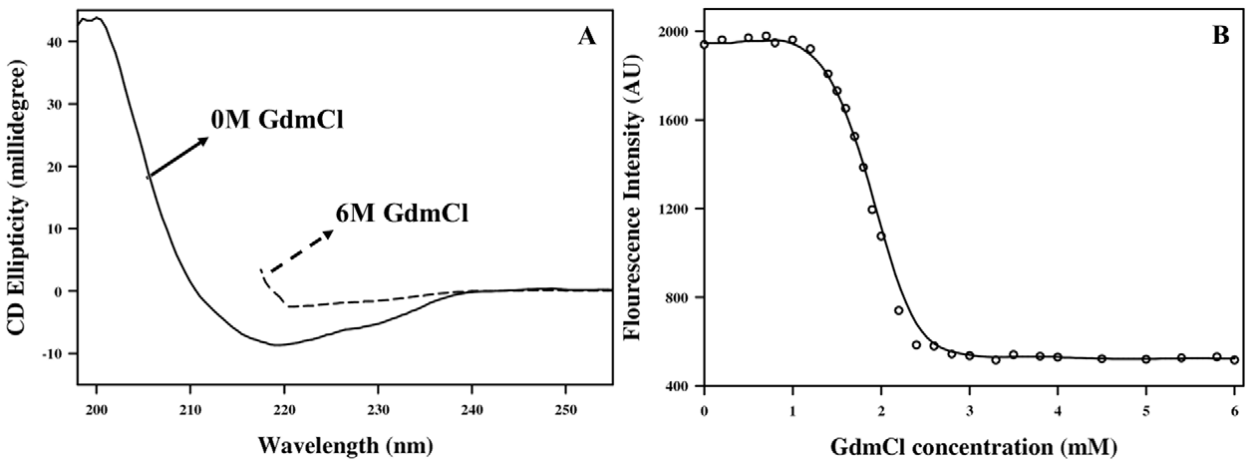
GdmCl dependent denaturation profile of M-crystallin. (A) Far-UV CD spectra of M-crystallin at pH 5.5 and 25°C, in the absence of GdmCl (0 M, solid line) and in presence of GdmCl (6 M, dotted line). (B) Fluorescence spectra of GdmCl-induced unfolding of M-crystallin. The line through the points is the fit to the two-state denaturation model. Protein was used at a concentration of 0.2 mg ml^−1^ in 50 mM Tris–Cl (pH 5.5) buffer containing 50 mM NaCl.

GdmCl induced unfolding of M-crystallin was characterized by CD and fluorescence emission spectra [32], [33], [34] as described in Materials and Methods. Figure 2 shows the denaturation curves of M-crystallin as obtained by far-UV CD and fluorescence spectroscopy, wherein the ellipticity at 218 nm (Figure 2A) and the Trp emission at 331 nm (Figure 2B) were monitored, respectively. The normalized fluorescence and CD curves (not shown here) show similar trend. Almost no changes were noticed till the GdmCl concentration reached 1.1 M. Between 1.1 and 2.8 M, there was a sudden change, with no change thereafter (Figure 2B). As it is evident from the Figures 2A and 2B, both the curves depict protein unfolding as a two-state model. Transition mid-point and slope of the transition (m_1_) thus determined from the fluorescence data were 1.9 M and 2.84 kCal mol^−1^ M^−1^, respectively.

### GdmCl dependent [^15^N-^1^H]-HSQC spectra

We used NMR to study the order-disorder transitions in M-crystallin under various denaturing conditions ranging from 0 to 6 M GdmCl. For this purpose, we recorded a set of [^15^N-^1^H]-HSQC of M-crystallin taken in 0, 0.8, 1.7, 2.0, 3.1, 4.0, 5.2 and 6.0 M GdmCl (Figure S2). As evident from Figure 1 and Figure S2, the protein was in a completely folded state at 0 M GdmCl concentration, with a single set of peaks. However, we started observing two sets of peaks as the GdmCl concentration approached 2 M (Figure S2), one set of peaks corresponding to the folded state (well dispersed peaks; as seen at 0 M) and the other set corresponding to a partially unfolded state (with the ^1^H^N^ chemical shifts in a narrow range of chemical shifts; between 7.8–8.8 ppm). This indicated that the protein was not in a completely unfolded state at the above-mentioned denaturant concentration. This observation of protein in its two states, correlates with the biophysical data described above. Further, as the denaturant concentration approached 3 M, the protein showed spectral signatures largely expected from the completely unfolded state of the protein. At this concentration and beyond, only one set of ^15^N-^1^H^N^ peaks showed-up with the corresponding ^1^H^N^ chemical shifts in a narrow range of 7.8–8.8 ppm. This observation is consistent with the fluorescence data discussed above, which indicates loss of the tertiary structure at higher GdmCl concentrations beyond 2.8 M. However, it is worth mentioning here that the protein showed some transient residual structures at denaturant concentrations greater than 3 M, which we probed and characterized, as discussed later.

### Structural preferences at different GdmCl concentrations and its comparison with folded protein

#### (a) Secondary structure chemical shift preferences (ΔC^α^-ΔC^β^) and *^3^J(^1^H^N^-^1^Hα)*

**Figure 3:**
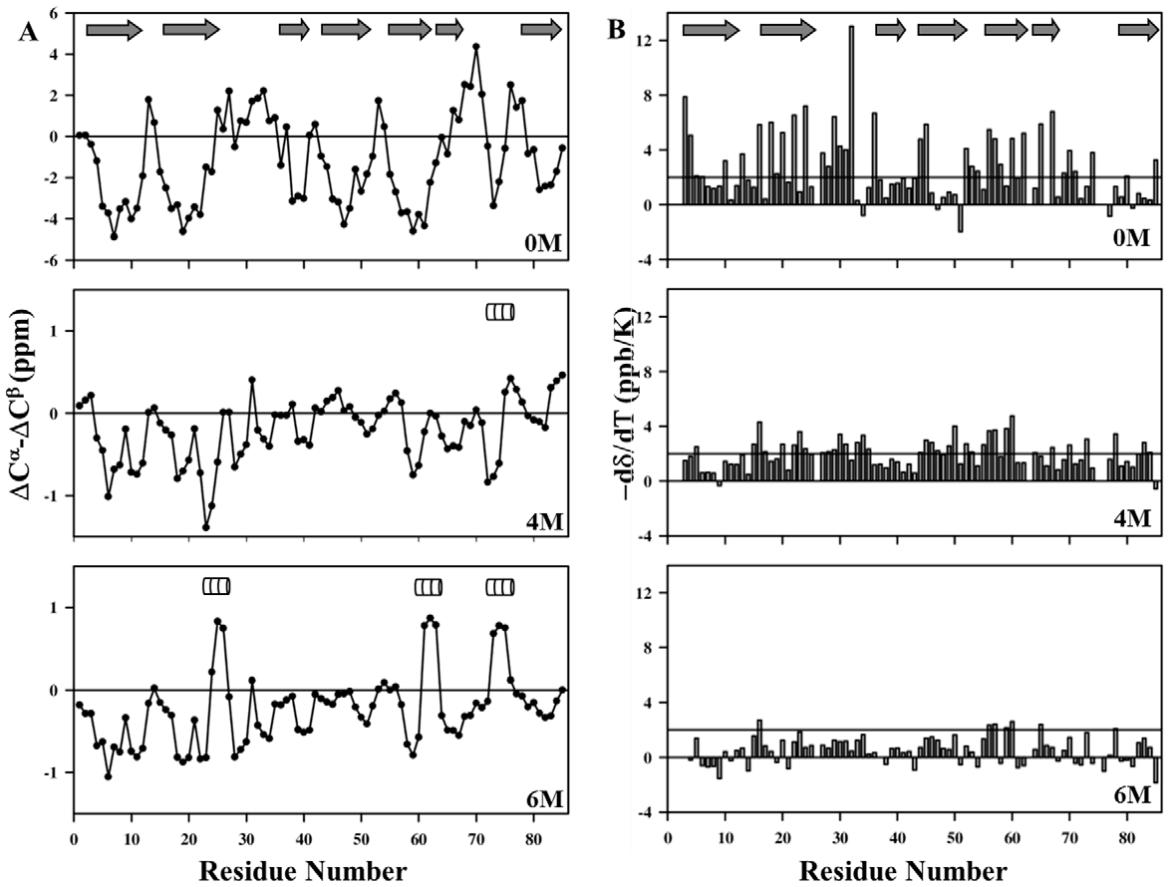
Secondary structure elements and temperature coefficients of M-crystallin. (A) Plot of secondary chemical shifts (ΔC^α^-ΔC^β^) for M-crystallin with 4 and 6 M GdmCl concentrations, and their comparison with that of the folded monomer. New structural elements seen are shown with boxes. (B) Residue-wise temperature coefficients of M-crystallin taken in 0, 4 and 6 M GdmCl concentrations. The bar line was fixed at a value +2. Residues representing the secondary structure elements in folded case (0 M) are shown as arrows at top of the both the plot panels A and B.

The secondary structural preferences of M-crystallin in 4 and 6 M GdmCl were estimated from the analysis of empirical relations of ^13^C chemical shifts (ΔC^α^-ΔC^β^) [35] (shown in Figure 3A) and *^3^J(^1^H^N^-^1^Hα)* three-bond coupling constants (Figures S3 and S4). While, the folded protein has a rigid structure, which is predominantly β-sheet in structure, and made up of seven well defined β-strands (Val 6-Glu 10, Ser1 8-Ala 21, Ser 38-Val 41, Thr 45-Tyr 49, Trp 59-Gly 62, Gly 64-Tyr 66 and Ser 81-Gln 84), the protein under denatured condition shows similar secondary structural propensities in these regions. As discussed earlier by several researchers, the denatured state(s) of any given protein may either adopt a completely random coil conformation [36], [37], [38] or may have regions which adopt preferred conformations or secondary structure propensities for transient structure formations [39], [40], [41]. The regions, which have certain secondary structural propensities, are termed as folding cores. These folding cores indicate a possible initiation of the folding reaction upon dilution of the denaturant concentrations. Figure 3A suggests that M-crystallin retained its predominantly β-sheet conformation during its unfolding pathway. However, three polypeptide stretches (Asp 20-Ala 28, Asp 60-Tyr 66 and Ala 72-Asn 77) did show propensities for helical conformation for the M-crystallin in 6 M GdmCl.

As mentioned below in Materials and Methods, *^3^J(^1^H^N^-^1^Hα)* were calculated from GFT (3, 2)D-HNHA spectra. We could measure *^3^J(^1^H^N^-^1^Hα* values for 77 out of 80 residues (>95%) in the native state of the protein, while the number of ^3^J values were 75 and 68 for the M-crystallin in 4 and 6 M GdmCl (Figure S4). These ^3^J couplings are highly sensitive to the backbone torsion angle φ and carry the information about the conformational preference of individual amino acid residues. The *^3^J(^1^H^N^-Hα)* for residues in α-helical segments (α-helix and PP_∥_) range between 4.0 and 5.5 Hz, while theses values are >8 Hz for residues involved in β-strand structures and 5.5–8.0 Hz for random coil stretches. For the native state of M-crystallin (2k1w), while ∼3% residues adopt an α-helical conformation, 42% were in β-sheet and 55% in random coil conformation. In the presence of GdmCl, most of these couplings range between 5.5 and 8.5 Hz, indicating their involvement in an extended structure with some kind of conformational averaging. However, for the polypeptide stretches, Thr22-Gln25, Gly64-Ser68 and Glu70-Ile74, the measured coupling constants were less than 5 Hz indicating α-helical or PP_∥_ preferences for these stretches.

#### (b) Temperature coefficients and residual structure formation

Amide proton (^1^H^N^) exchange rates and their temperature coefficients throw light on hydrogen bonding (if any), both in globular or denatured state of any protein [42]. If an ^1^H^N^ is hydrogen bonded, it would have a temperature coefficient more positive than −4.5 ppb/K. Random coiled or non-hydrogen bonded ^1^H^N^ tends to have more negative values than −4.5 ppb/K. In the present study, the ^1^H^N^ temperature coefficients were measured by recording a set of HSQC spectra at different temperatures ranging from 15 to 36°C, at an interval of 3°C, for all the three states of the protein (Figure 3B). As seen from Figure 3B, the protein in its native state (0 M GdmCl) displayed the presence of several continuous polypeptide stretches involved in hydrogen bonding. On the other hand, the protein under denatured conditions (4 M and 6 M GdmCl) exhibits largely extended conformations, with very few signatures of intramolecular hydrogen bonding. The polypeptide stretches that showed signatures of their involvement in hydrogen bonding were Thr22-Asp24, Glu65-Tyr66, and Asn77-Ser78. These polypeptide stretches showed temperature coefficients more positive than −4.0 ppb/K values even in 4 M GdmCl, hinting at their possible involvement in some kind of residual structures even under such denatured conditions.

### Protein dynamics in the denatured states

The ^15^N spin-lattice/spin-spin relaxation times (T_1_/T_2_), and heteronuclear ^1^H-^15^N Overhauser effects (NOE) were measured at 25 °C and at two different external magnetic fields (600 and 800 MHz) for the folded protein as well as the protein in the denatured states to throw light on GdmCl induced motional perturbations. We carried out relaxation analysis of 69 and 65 residues of the protein in 4 and 6 M GdmCl, respectively. Relaxation data for the folded protein was taken from our earlier reported work [26]. Three prolines and two N-terminal residues, which do not show their spectral signatures could not be part of this dynamic study either in the native or the denatured state of the protein.

#### (a) Relaxation parameters

**Figure 4:**
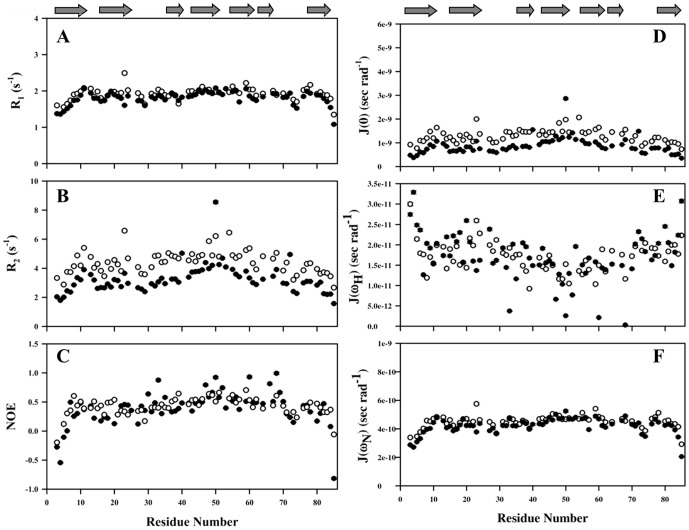
^15^N relaxation parameters and reduced spectral densities versus residue number. (A) Longitudinal relaxation times (T_1_). (B) Transverse relaxation times (T_2_). (C) [^1^H-^15^N] NOE enhancements are defined as I_sat_/I_eq_ where I_sat_ and I_eq_ are the intensities of peak in the 2D experiments with and without proton saturation described above, respectively. Error bar of the T_1_ and T_2_ data denote curve-fitting uncertainties: errors in the [^1^H-^15^N] NOEs are estimated from the signal/noise ratio of the spectra. Spectral densities are shown in (D) J(0), (E) J(ω_H_), and (F) J(ω_N_), as a function of protein sequence number. Here, filled and open circles correspond to the 4 and 6 M GdmCl cases, respectively. Secondary structure elements seen in the folded protein (0 M) are depicted at the top of both the plots.

The longitudinal relaxation (R_1_) rates are highly field dependent and are sensitive to nanosecond-picosecond time scale motion. R_1_ remained constant across the polypeptide stretch with average values of 1.9±0.1, 1.78±0.06 and 2.65±0.1 s^−1^ for the protein in 6, 4 and 0 M GdmCl, respectively (Figure 4A and Table 1). Both termini of the protein expectedly showed lower values of R_1_ suggesting faster time scale motions at both ends. Significantly, large values of R_1_ is seen for residues Asn 31, Lys 35, Ser 38, Tyr 51, Gly 55, Ser 68, Ser 71, and Ile 79 at 0 M; His 11, Glu 50, Asn 53, Asn 77, and Ser 78, at 4 M and Ser 23, Asp 24, Gly 27, Trp 59 and Ser 78 at 6 M GdmCl concentrations (Figure 4A and Figure S5A).

**Table 1:** Comparison of Backbone Dynamics Parameter for greek-key1 and greek-key2 in M-crystallin.

	0 M GdmCl	4 M GdmCl	6 M GdmCl
**Motional parameters for residues with secondary structures**			
Greek key 1			
Average R1(s)	2.67 (±0.09)	1.79 (±0.09)	1.85 (±0.10)
Average R2(s)	7.11 (±0.37)	3.13 (±0.21)	4.36 (±0.17)
Average NOEs	0.71 (±0.03)	0.35 (±0.05)	0.45 (±0.04)
Average S^2^	0.83	ND	ND
Greek key 2			
Average R1(s)	2.68 (±0.08)	1.81 (±0.08)	1.91 (±0.11)
Average R2(s)	6.69 (±0.41)	3.36 (±0.19)	4.33 (±0.21)
Average NOEs	0.73 (±0.03)	0.42 (±0.06)	0.43 (±0.05)
Average S^2^	0.81	ND	ND
**Average R_2_ (and nOe) of β-strands and loop** **Greek key1 vs Greek key 2**			
β1 (6–9) vs. β5 (44–50)	9.19(0.55)**/**8.45(0.61)	2.75(0.26)**/**4.64(0.63)	4.12(0.42)**/**5.19 (0.54)
β2 (18–21) vs. β6 (59–61)	7.28(0.58)**/**8.99(0.64)	3.00(0.28)**/**3.25 (0.60)	4.30 (0.45)**/**4.72 (0.54)
β4 (38–41) vs. β8 (80–85)	9.53(0.55)**/**7.56(0.59)	3.77(0.40)**/**2.35 (0.08)	4.73 (0.59)**/**3.51 (0.30)
β7 (64–66) vs. β3* (27–30)	7.09(0.66)**/**8.64(0.56)	3.24(0.64)**/**2.53 (0.38)	4.82 (0.38)**/**3.75 (0.29)
loop a (10–16) vs loop b (51–56)	7.34(0.88)**/**7.87(0.91)	3.19(0.34)**/**3.86 (0.49)	4.42 (0.43)**/**4.93 (0.55)
**Calcium binding region1 (31–36)**	10.89 (0.84)	3.00 (0.51)	4.61 (0.41)
**Calcium binding region2 (76–80)**	10.88 (0.88)	3.00 (0.34)	4.11 (0.44)

* -not a β-sheet in the NMR structure. ND- not determined. Calcium binding region 1 is annotated as loop-1 and calcium binding region 2 is annotated as loop-2.

The transverse relaxation (R_2_) rates are important to get information about the conformational transition in the millisecond to microsecond time-scale regime. They showed larger variation all along the sequence ranging from 2.66 to 6.58 s^−1^, 1.56 to 8.54 s^−1^ and 5.01 to 14.18 s^−1^ for the protein in 6, 4 and 0 M GdmCl (Figure 4B). The polypeptide stretches Val 8-Phe 14, Asp 20-Gln 25, and Lys 42-Arg 57 showed higher R_2_ values (>6 s^−1^) for the protein in 4 and 6 M GdmCl concentrations. Here, Ala 21 and Ala 28 show lower values of R_2_ compared to their respective values seen in the native state of the protein [26]. These residues, belonging to a different Greek keys, are seen to have a higher value of exchange and R_2_ value in case of native protein [26].The N- terminal residues and Lys 17, Ala 28, Gly 29, Gly73, and Ile74 residues also show lower R_2_ values due to their presence at the edge of structural propensities. This is also supported by the fact that these residues are in the neighborhood of residues Gly, Pro or Ala, which are supposed to create hinges with in a polypeptide stretches and/or act as secondary structure breakers.

The ^1^H-^15^N steady state nOes have information about high frequency motions As is evident in Figure 4C, three N-terminal residues Asn 3, Ala 4 and Glu 5, and two C-terminal residues Gln 84 and Ile 85 show negative nOes, as a result of their higher order flexibility (Figure 4C and Figure S5C) in the presence of denaturant (6 and 4 M GdmCl), while the protein in its native state (at 0 M GdmCl) does not show such higher order flexibility at the C-terminal end. All the relevant R_1_, R_2_ and nOe data are provided in Table 1.

#### (b) Spectral densities

^15^N-relaxation data acquired on 600 MHz NMR spectrometer were used to calculate the reduced spectral density functions (J values) at different frequencies (0, 60 and 540 MHz). These J(ω_H_), J(ω_N_) and J(0) values were calculated based on Equations 2–5 (see Methods; Figure 4D–F). The J(0) values showed the largest variation across the sequence and are mostly based on R_2_, whereas the J(ω_H_) values, which are largely determined by nOe data, exhibited a lot of variation in the regions belonging to terminal residues, Asp 20-Ala 28, Gly 44-Ile 52, Asp 60-Tyr 66 and Ala 72-Asn 77. The J(ω_N_) values, which are largely dependent on R_1_, were found to be constant for most of the residues, except for regions at the N and C-terminals, and the polypeptide stretches Thr 22-Ala 28, and Gly 73-Ser 80. A higher degree of variation was observed for the terminal residues at both 4 and 6 M concentrations of the denaturant. In the native state, large variations in J(ω_H_) and J(ω_N_) values were seen for polypeptide stretches Ala 28-Asn 33 and Asn 77-Arg 83. These residues showed higher J(0) values and have chemical exchange contribution under native state [26]. The calculated average values of J(0), J(ω_H_) and J(ω_N_) were 2.96 ns rad^−1^, 4.35 ps rad^−1^ and 0.539 ns rad^−1^, respectively at 0 M concentration, whereas they were 0.864 ns rad^−1^, 17.08 ps rad^−1^, 0.421 ns rad^−1^ at 4 M and 1.29 ns rad^−1^, 17.09 ps rad^−1^, 0.449 ns rad^−1^ at 6 M concentration, respectively (Figure 4 D–F. Figure S5 D–F)).

#### (c) Correlation times

**Figure 5:**
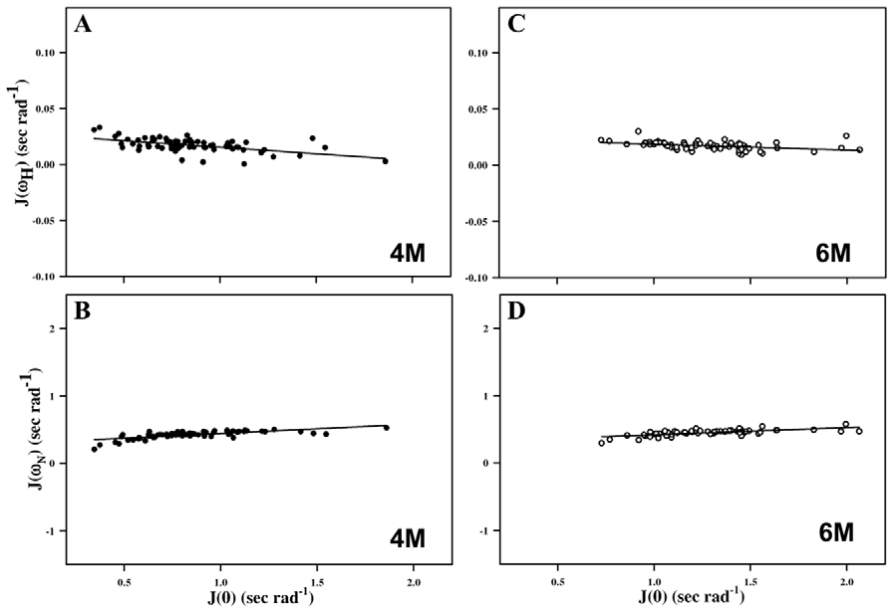
Correlation between spectral density functions. Plot of J(ω_H_) versus J(0) (A, C) and J(ω_N_) versus J(0) (B, D). The fits were obtained by linear regression, J(ω_N,H_) = α J(0)+β. Here α is the slope and β is the intercept on ω_H_/ω_N_ axis. Filled circled (A, B) plots denote M-crystallin taken in 4 M GdmCl concentration, whereas open circled (C, D) plots correspond to the protein taken in 6 M GdmCl concentration.

The correlation time provides details into different time-scale motions observed in a polypeptide chain. The linear correlation between *J*(ω*_N,H_*) and corresponding *J(0)* values using the Equation J(ω_N,H_) = αJ(0)+β, and Equation (6) were used to calculate correlation times under different denaturing conditions (Figure 5 A–D). The calculated correlation times were 4.67, 1.39 and 1.68 ns for the native protein (0 M GdmCl); 3.54, 1.09, 1.28, 4.42, and 0.071 ns for the protein in 4 M GdmCl, and 3.97, 1.01, 1.27, 10.06 and 0.062 ns for the protein in 6 M GdmCl (Table 2). These values clearly depict different time-scale motions under different denaturant conditions. The correlation time 10.06 ns associated with the protein in 6 M GdmCl corresponds to global correlation and this value is double the value obtained for the natively folded protein. This is probably due to the following reasons: (i) complete unfolding of the protein, (ii) higher viscosity, as seen in the presence of 6 M GdmCl, and (c) intermolecular association under unfolding conditions [43], [44]. The shorter correlation times 1.01, 1.27, and 0.062 ns reflect independently fluctuating segments of the protein in 6 M GdmCl whereas 3.97 ns corresponds to the overall tumbling time of M-crystallin in its native form (Table 2).

**Table 2:** All tumbling times (in ns) at various GdmCl concentrations are given in the table.

GdmCl conc.	xN1	xN2	xN3	xH1	xH2	xH3
0 M	**4.67**	1.39	IM**	*79.30* *	**1.68**	0.042***
4 M	**3.54**	1.09	IM	1.28	**4.42**	0.071
6 M	**3.97**	1.01	IM	1.27	**10.06**	0.062

* -sub-µs motion,

** -imaginary value,

*** -ps motion.

These measurements were done at 25°C. The equation *2αω_(H,N)_^2^τ_c_^3^+5βω_(H,N)_^2^τ_c_^2^+2(α−1)τ_c_+5β = 0* was used for the calculation. xHs/xNs represent the respective roots for proton (using ω_H_) and nitrogen (using ω_N_).

#### (d) Conformational exchanges (R_ex_) contribution

**Figure 6:**
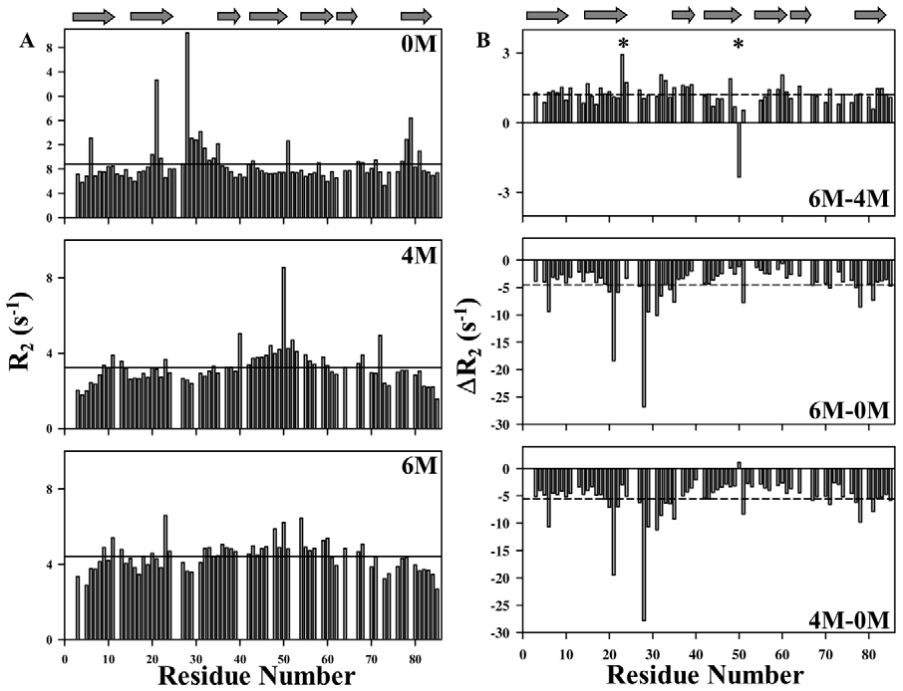
Transverse relaxation rates (R_2_) in M-crystallin. Left panel (A) represents measured R_2_ values at 6, 4 and 0 M GdmCl concentrations against protein sequence. Right panel (B) shows the deviations in R_2_ values for 6, 4 and 0 M GdmCl. Horizontal lines in each box indicate average values. Residues Asp 24 and Glu 50 are marked with asterisks.

The R_2_ values are representative of slow time-scale motions including conformational exchanges. They provide evidence for motional restrictions and flexibilities in native as well as in denatured proteins. The R_2_ values determined at different denaturing conditions are shown in Figure 6A. As evident in Figure 6A, R_2_ values showed finite variations all along the protein primary sequence indicating some degree of restricted motions even under denaturing conditions. Figure 6B shows the changes in the R_2_ values for the protein in going from 0 to 4 M, 0 to 6 M and 4 to 6 M of GdmCl concentrations. The negative values in Figure 6B represent increased conformational transitions, while positive deviations indicate decreased conformational transitions. These features were predominantly seen for residues Asp 24 and Glu 50, while going from 4 to 6 M GdmCl concentration. Here, Asp 24 shows decreased conformational transition whereas Glu 50 exhibited increased conformational transition (Figure 6B). Further, as is evident from Figure 6 A/B, Val 8, Thr 22, Ala 8 and Ser 78 were observed to have increased conformational exchange in its native state (0 M GdmCl) as compared to the protein with 6 M GdmCl. This could be attributed to the fact that, as the protein attains its native fold, the mechanism for conformational exchange is driven by several factors such as change of viscosity, presence of stable secondary structural elements etc [43], [44].

**Figure 7:**
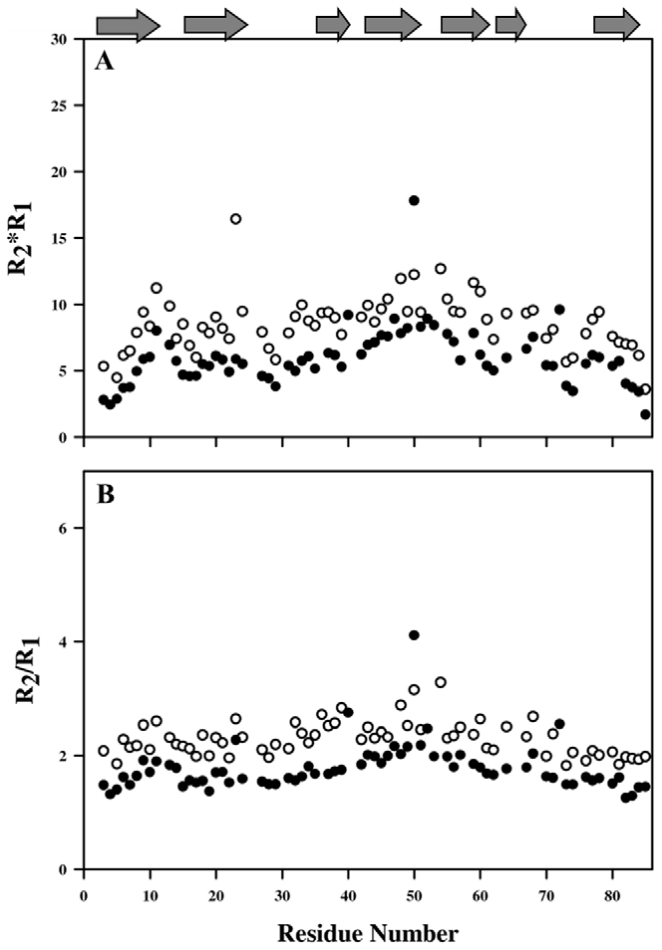
Conformational exchange in M-crystallin. Plots depicting the conformational exchange for the protein taken in 4 M (filled circles) and 6 M (open circles) GdmCl. (A) R_2_×R_1_ and (B) R_2_/R_1_.

Conformational exchange contributions (R_ex_) to the R_2_ were calculated based on equation R_2_*R_1_ and R_2_/R_1_ [45], [46]. Higher values of R_2_/R_1_ ratio represent the presence of conformational exchange and shed light on motional fluctuations (Figure 7A/B). Conformational exchange is expected to increase with higher-field strength (not shown in this study). Significantly large conformational exchange contributions were observed for residues Tyr 9, Ala 21, Gln 25, Ala 28, Asn 31, Leu 32, Lys 40, Thr 45, Leu 61, Ser 71 and Ile 78 for the protein with 0 M GdmCl concentration; residues His 11, Glu 50, and Ala 72 at 4 M GdmCl, and residues His 11, Ser 23, Tyr 54 and Ser 78 at 6 M GdmCl. These residues are present either at the edge of the secondary structure in M-crystallin or in the loop region of the protein.

### Residual structural preferences in the unfolded states

**Figure 8:**
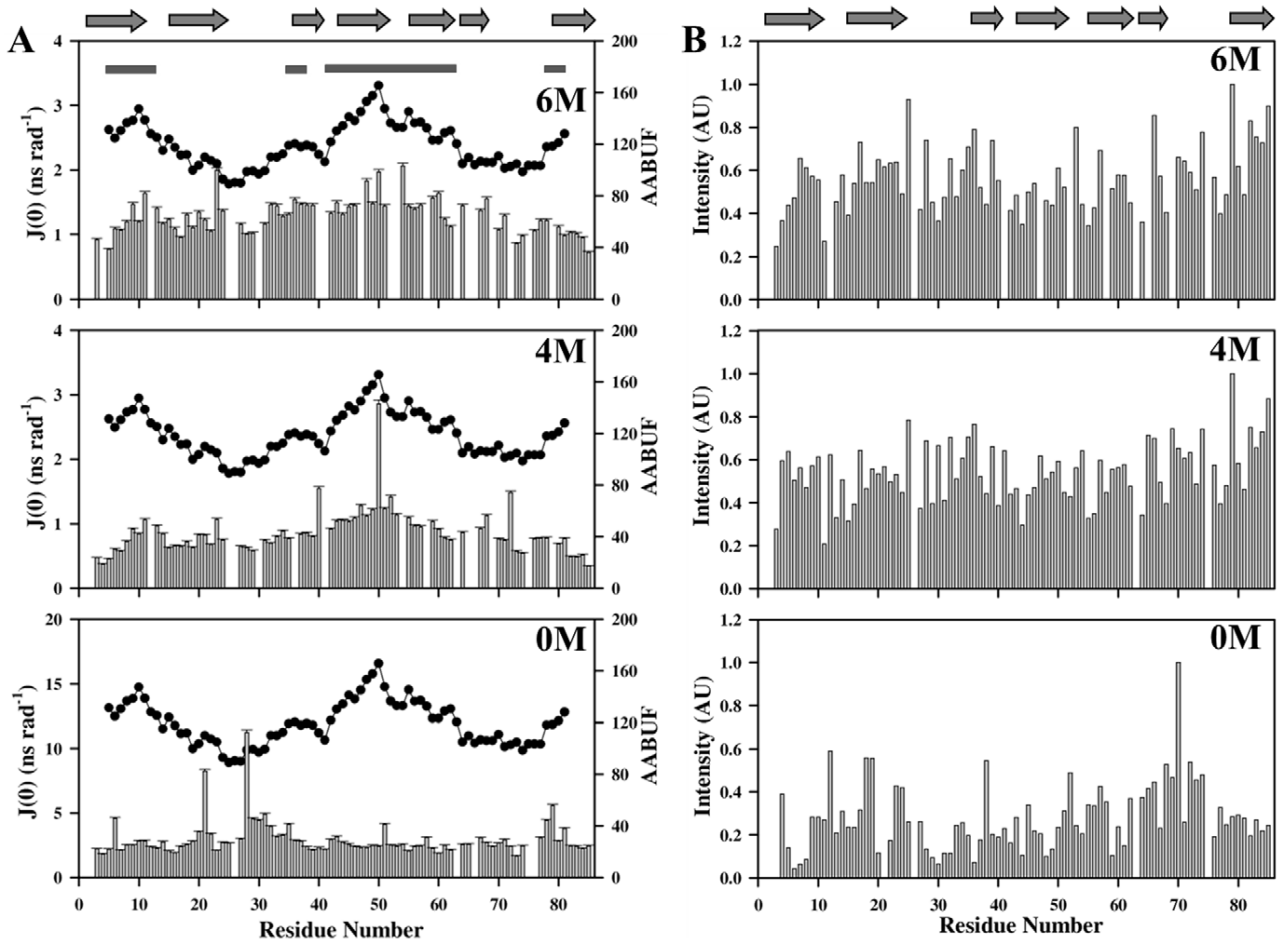
Spectral density, AABUF and normalized cross-peak intensity analysis. (**A**) Plots of J(0) values (vertical bars with error) and AABUF (Average Area Buried upon Folding) (filled black circles). Horizontal bars on top of the plot indicate the regions with AABUF value more than the average. (B) Normalized cross-peak intensities derived from 2D [^15^N-^1^H]-HSQC of M-crystallin at different denaturant concentrations. The folded secondary structural elements (0 M GdmCl) are shown on the top of panel.

The presence of hydrophobic clustering has been quite often reported to be the prime reason for the formation of residual structural element(s) in unfolded state of proteins [38], [47], [48], [49]. The hydrophobic clustering in M-crystallin (if any) was quantified using the per-residue average area buried upon folding AABUF [50], which is defined as the “change in average area of each amino acid residue upon unfolding”. AABUF is well correlated in several unfolding studies [51], [52], [53] and is used as a measure for the identification of folding sites in proteins. The correlation of J(0) values and AABUF with the protein primary sequence is shown in Figure 8A. As is evident from this figure, the AABUF values were relatively higher in the polypeptide stretches Val 6-Phe 14, Lys 42-Gly 62, and Ser 78-Phe 82. Interestingly, J(0) and the AABUF showed a similar trend in these regions only under denaturing conditions of 4 and 6 M GdmCl. These regions with reduced flexibilities identified from J(0) analysis were found to correlate well with increased AABUF values (increased hydrophobic content). This correlation suggests the presence of local hydrophobic clustering in these regions under denaturing conditions. A further analysis of HSQC peaks with lower intensities or absence of HSQC peaks in these regions led to our conclusion of occurrence of possible transient hydrophobic clustering under denaturing conditions mentioned above (Figure 8B). It is also interesting to note that region Asn 77-Arg 83 shows variation in J(ω_H_) and J(ω_N_) values in its native state (0 M GdmCl). Incidentally, the three polypeptide stretches Val 6-Phe14, Lys 42-Gly 62, and Ser 78-Phe 82 discussed above comprise four β-strands, namely β1, β4, β5 and β7, in the native state protein structure (Figure 8A). Thus, it is obvious that local interactions in these regions resulted in restricted time scale motions, which can result in the formation of residual structures, especially in the regions Thr 22-Ala 28, Asp 60-Tyr 66 and Ala 72-Asn 77. This is in line with the α-helical propensities of these residues under 6 M GdmCl concentration.

Furthermore, we recorded [^15^N-^1^H]-HSQC-NOESY spectra with different mixing times (τ_m_ 100 and 140 ms) to look for medium- and long-range nOes (if any) in the NOESY spectra of the protein under different denaturant conditions. Unlike the native state protein, which showed several medium- and long-range nOe connectivities, the protein under different GdmCl concentrations did not show any long-range nOes, except few d_NN_(i, i+1) connectivities, which are commonly expected in α-helical segments, along the polypeptide stretches Asp 20-Gln 25, and Ser 68-Gly 73, of the protein in 6 M GdmCl (Figure S6). These ^1^H-^1^H nOe connectivities throw light on the structural preference of these corresponding polypeptide stretches. These observations, taken together, support the propensity of both α-helical and β-strand structural elements of the M-crystallin under denaturing conditions.

### Sequence-structure-function paradigm and folding mechanism

The primary sequence, structure and dynamics reflect the design of function of any protein. Structural Biology is guided by this motivation. Solution structures are ideally suited for unraveling such functional states. It is widely accepted that residual structures under denaturing conditions are indicative of structural nucleation for proper folding of the protein [41]. For M-crystallin under different denaturing conditions, many segments followed the β-strand structural preferences, with few polypeptide stretches showing α-helical propencities, Based on the temperature coefficients, dynamics study and nOe information, we propose a model for the folding of M-crystallin. The protein starts from the natively folded state when the M-crystallin is in 0 M GdmCl [25]. Subsequently, protein undergoes transition and starts showing two distinct sets of peaks when it is in the 2 M GdmCl (Figure S2). Thereafter, when the protein is in 4 M GdmCl, it starts showing secondary structural elements and finally adopts an unfolded state under 6 M GdmCl.

### Unfolding Study of M-crystallin and its biological relevance

The unfolding study of M-crystallin under different GdmCl concentrations provided residue level insights into the intrinsic conformational preferences of different polypeptide stretches in the protein. The backbone dynamics, NOESY data and biophysical data of the protein under different GdmCl concentrations taken together suggest that the protein adopts few α- and β-type structural preferences under different denaturing conditions. In conclusion, these fluctuating structural preferences and the possible intermolecular interaction/association, as predicted based on the spectral density data, might be a leading cause for aggregation of crystallins resulting in cataract.

## Materials and Methods

### Protein sample preparation

^13^C- or/and ^15^N-labelled M-crystallin were over-expressed and purified as mentioned elsewhere [16] [54]. The purity of samples was checked using SDS-PAGE and mass spectroscopy (MALDI-TOF). The protein samples denatured in the presence of varying concentrations of *guanidine-HCl* (GdmCl; in 0–6 M range at an interval of 0.2–0.3 M) were prepared in milliQ H_2_O solution (10 mM Tris, 50 mM KCl, 5–10 mM CaCl_2_, pH 5.5) for various purposes as discussed above.

For NMR measurements, the protein samples were concentrated to 1.2 mM and exchanged with the proper buffer containing GdmCl at concentrations 0, 1, 2, 4, and 6 M. NMR experiments were recorded after equilibrating the samples in GdmCl for 6–8 hrs. The data acquired with the protein samples in 4 and 6 M GdmCl concentrations is discussed in the results and discussion.

### Denaturation studies in guanidine-HCl

The denaturation profile of M-crystallin was studied by optical (circular dichroism (CD) and fluorescence) and NMR spectroscopy. The GdmCl concentrations were determined using a refractometer. For optical measurements, the protein samples of 45 µM concentration with different concentrations of GdmCl were prepared and equilibrated for at least a period of 5–8 hrs.

Far-UV CD spectra were recorded at 25°C on a JASCO J-810 spectropolarimeter (JASCO, Europe) and corrected for buffer baseline. Each scan ranged from 200 to 260 nm with a scan speed of 20 nm/min with a quartz cell having a path length of 0.1 cm. The CD spectra below 200 nm were saturated due to high salt (particularly in the presence of 6 M GdmCl) and hence were not included in the data analysis. Each spectrum was an average of 4 scans (Figure 2A).

Fluorescence spectroscopy experiments were performed on a Hitachi F-4500 spectrofluorimeter with a protein concentrations of 45 µM, as mentioned above (Figure 2B). The Trp emission spectra were recorded by exciting the protein samples at 295 nm. The intensity maxima, wavelength maxima, and intensity value at 331 nm were plotted as a function of GdmCl concentration to determine the fractions unfolded. The data were fitted to a two-state denaturation model, and parameters were determined by a non-linear curve fitting to the following equation [55]:
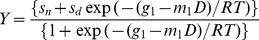
(1)where, *Y* is the observed spectroscopic signal; *s*_n_ and *s*_d_ represent the spectroscopic signals of folded and denatured proteins, respectively; *g*_1_ and *m*_1_ represent the free-energy change and slope of the transition, respectively; *D* is the denaturant concentration; *T* is the temperature (in K), and *R* is the universal gas constant (1.987 cal K^−1^mol^−1^).

### NMR spectroscopy

NMR experiments were performed either on a Bruker Avance spectrometer with a ^1^H frequency of 800.12 MHz, or on a Varian Inova spectrometer with ^1^H frequency of 600.51 MHz at 25°C. Both the spectrometers were equipped with cryogenically cooled probes. Sensitivity-enhanced 2D [^15^N-^1^H]-HSQC was recorded for each NMR sample, with 256 and 1024 complex data points along the ^15^N and ^1^H dimensions, respectively. The spectral widths along ^1^H and ^15^N dimensions were 12 and 30 ppm, respectively. No changes in HSQC spectra were noticed during the data acquisition, indicating a stable equilibrium under all the experimental conditions. The data were apodized using a sine-squared bell window functions along both the dimensions, zero filled to yield a final resolution of 0.9 and 2.5 Hz/pt along ω_1_ and ω_2_ dimensions, respectively, and followed by Fourier transformation. A suite of 3D experiments (CBCACONH, CBCANH, HNCO) and ^15^N-edited TOCSY-HSQC with a mixing time of 80 ms were recorded for complete resonance assignments [27], [28]. ^15^N-and ^13^C-edited NOESY-HSQC, with a mixing time of 100 and 140 ms, were recorded to get information about ^1^H-^1^H nOes. Number of scans were 8–24 scans for different experiments, with 32–64 and 48–80 complex increments along ^15^N and ^13^C dimensions, respectively. 100–128 complex increments were used along the indirect ^1^H dimension.

Relaxation data were recorded on both 600 and 800 MHz spectrometers at 25°C [56], [57]. ^15^N-spin lattice relaxation rates (R_1_ = 1/T_1_) were measured with inversion recovery delays of 10, 30*, 50, 70, 90*, 130, 240, 410*, 610, 860, and 1100 ms, where the delays marked with an asterisk were recorded twice. ^15^N-spin-spin relaxation rates (R_2_ = 1/T_2_) were measured with Carr-Purcell-Meiboom-Gill (CPMG) delays of 10*, 30, 50*, 70, 90, 110*, 130, 150, 170, and 190 ms. Steady state [^15^N-^1^H]-heteronuclear nOe measurements were carried out with the proton saturation time and relaxation delay of 3 s each. A relaxation delay of 6 s was used in the experiment without proton saturation. 32 scans were used during R_1_ and R_2_ measurements, while 48 scans were used for the heteronuclear nOe measurement.

Amide proton temperature coefficients were measured by recording a suite of sensitivity-enhanced 2D [^15^N-^1^H]-HSQC at different temperatures ranging from 15 to 36°C, at an interval of 3°C, with 256 t_1_ increments along the ^15^N dimension. Number of scans was 16, and the spectral widths were 12 and 30 ppm along the ^1^H and ^15^N dimensions, respectively. The protein was stable over the entire temperature and pH (5.5 and 7.5) ranges. ^3^J(^1^H^N^-^1^Hα) coupling constants were measured from (3, 2) HNHA [31]. All the experiments were processed using Felix (Accelrys Software Inc., San Diego, CA), NMRPipe [58] and analyzed using Felix (http://www.felixnmr.com) and CARA (http://cara.nmr.ch). The (3, 2)D HNHA, (3, 2)D HNHB, (3, 2)D CB(CACO)NHN, (3, 2)D CT-HCCH-COSY spectral data were processed and used as mentioned elsewhere [31]. Sensitivity-enhanced 2D [^15^N-^1^H]-HSQC were recorded at the beginning and end of all the 3D experiments and compared to make sure the stability of the protein. No observable changes were seen in the HSQC indicating high stability of the protein during the entire collection of the NMR Data. All the data were apodized with a sine-bell window function, shifted by 56° along both the dimensions of 2D data, and by 60° along all the three dimensions of 3D data, followed by zero-filling and Fourier transformation. The final processed data matrices had 2048*1024 and 1024*128*256 complex data points in all 2D and 3D spectra, respectively.

In the temperature coefficient measurement study, the chemical shift information derived from individual HSQC was tabulated and fitted to a straight line and the corresponding temperature coefficients (dδ/dT) were determined from its slope. The digital resolutions in these HSQC spectra were 2.0 and 0.5 Hz/pt along ω_1_ and ω_2_ dimensions, respectively.

In relaxation data measurements, peak heights with their respective errors were measured. The peak-heights thus derived were fitted to a single exponential decay function,
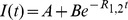
to derive the individual R_1_ and R_2_ values, where *I(t)* is the intensity at delay t (ms) used in the measurement of R_1_ and R_2_. A+B is the intensity at initial time t = 0, and A is the steady-state intensity at t = ∞.

The ^1^H-^15^N heteronuclear nOe was calculated from the following equation,
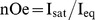
where, I_sat_ and I_eq_ are the intensities of individual peaks in the spectra recorded with and without proton saturation. The errors in the nOes were obtained using the root-mean-square value of the background noise as described by Farrow *et al.* [59], [60].

Proton chemical shifts were referenced using DSS at 0.00 ppm in H_2_O/^2^H_2_O solution at 25°C (6 M GdmCl, pH 5), whereas ^15^N and ^13^C were referenced indirectly as described elsewhere [61].

### Spectral density functions and correlation times

The spectral density functions *J(0)*, *J(ω_N_)*, and *J(ω_H_)* were calculated as described by Wagner *et al.* [59], [60] and Lefevre *et al.* [*62*] According to these approaches, *J(0)*, *J(ω_N_)*, and *J(ω_H_)* terms can be expressed in terms of ^15^N spin-lattice (R1) and spin-spin (R2) relaxation rates and heteronuclear [^1^H-^15^N] NOEs as follows:
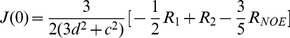
(2)

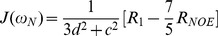
(3)

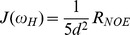
(4)where,
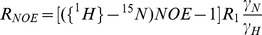
(5)The constant d^2^ is approximately equal to 1.35×10^9^ (rad/s)^2^, whereas the constant c^2^ is approximately 1.25×10^9^ (rad/s)^2^ and 2.25×10^9^ (rad/s)^2^ at 600 and 800 MHz, respectively. Errors in the individual spectral density functions were calculated from the error in the related parameters and by solving above equations [63]. After calculating *J(0)*, *J(ω_N_)*, and *J(ω_H_)*, the linear correlation between *J(0)* and *J(ω_N_)*, and *J(0)* and *J(ω_H_)* was examined (Figure 4).

Only few residues showed distinctly higher values of *J(0)*, indicating that chemical exchange motions have significant contributions to the mobility of their NH vectors. A linear correlation between *J*(ω*_N,H_*) and corresponding *J(0)* values using the equation J(ω_N,H_) = α J(0)+β, has been proposed for the calculation of τ_m_ [60], . The values of α and β derived from the plot of *J*(ω*_N_*) versus *J(0)* were found to be 0.14 and 0.30 ns/rad, respectively, for 4 M GdmCl case, whereas 0.11 and 0.31 ns/rad, respectively, for 6 M case. These values were then used to calculate the overall correlation time (τ_m_) using the following equation [60], [62]:
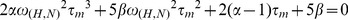
(6)


The solution of the above cubic equation yields three values of τ_m_ (Table 2). The values in milli-seconds to micro-seconds, sub-nano-seconds and pico-seconds represent the chemical exchange, overall rotational correlation time and internal motions, respectively, depending on their amplitudes.

### Average area buried upon folding

The per-residue average area buried upon folding (AABUF) was calculated using the method of Rose *et al.* [50]. A nine-residue moving-average window was used in the AABUF calculations.

## Supporting Information

Figure S1 **Sequence alighment of M-crystallin with other known lens crystallins, βB2-crystallin, γB-crystallin, ciona- crystallin and DdCAD, Trp corner and Tyr corner, special features of crystallins are shown with black arrow.** Higher sequence similarity in the alignment chart is highlighted with red color.
(TIF)

Figure S2 Sensitivity-enhanced 2D [^15^N-^1^H]-HSQC of [Ca^2+^]_2_-M-crystallin in the presence of 0 (A), 2 (B), 4 (C) and 6 M (D) GdmCl (pH 5.5 and temperature 298 K). These spectra were recorded on a Bruker Avance 800 MHz spectrometer with 128 and 1024 points along t_1_ and t_2_ dimensions, respectively. Individual peak assignments are shown by the corresponding single-letter code of the amino acid residue and its sequence number along the primary sequence.
(TIF)

Figure S3 (3, 2) D HNHA spectra of M-crystallin taken in (A) 4 and (B) 6 M GdmCl.
(TIF)

Figure S4 **^3^J(^1^H^N^-^1^Hα) verses residue number along the primary sequence, as measured from (3, 2) D HNHA for different GdmCl concentrations.** In (3, 2)D HNHA, couplings were measured using the formula mentioned in [31].
(TIF)

Figure S5 **^15^N relaxation parameters and reduced spectral densities versus residue number.** (A) Longitudinal relaxation times (T_1_). (B) Transverse relaxation times (T_2_). (C) [^1^H-^15^N] NOE enhancements derived from I_sat_/I_eq_ ratio, where I_sat_ and I_eq_ are the intensities of peaks in the 2D spectra recorded with and without proton saturation, respectively. Error bars in the T_1_ and T_2_ data denote curve-fitting uncertainties: errors in the [^1^H-^15^N] NOEs are estimated from the signal/noise ratio of the spectra. Spectral densities are shown in (D) J(0), (E) J(ω_H_), and (F) J(ω_N_), as a function of protein sequence number. Here, horizontal bars represent the data for the protein with 0 M GdmCl, filled and open circles correspond to the protein taken in 4 and 6 M GdmCl concentrations, respectively. Secondary structural elements of folded protein are depicted on top of the panels.
(TIF)

Figure S6 **NOE connectivities obtained from ^15^N-edited 3D NOESY-HSQC of M-crystallin taken in 6 M GdmCl (pH = 5.5; temperature = 25 °C).** The native secondary structure elements are depicted on top of the panel with arrows. Folded protein has 7 stretches of β-strands.
(TIF)
