# Supplementary figures and images for: Guanidine-HCl Dependent Structural Unfolding of M-Crystallin: Fluctuating Native State Like Topologies and Intermolecular Association
Source: PLoS One. 2012 Dec 17;7(12):e42948. doi: 10.1371/journal.pone.0042948 (PMC3524170; doi:10.1371/journal.pone.0042948)

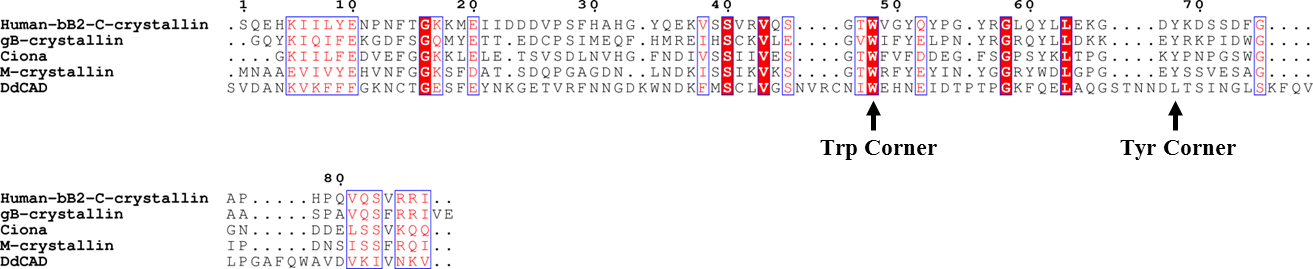

Supplement: Figure S1 — Sequence alighment of M-crystallin with other known lens crystallins, βB2-crystallin, γB-crystallin, ciona- crystallin and DdCAD, Trp corner and Tyr corner, special features of crystallins are shown with black arrow. Higher sequence similarity in the alignment chart is highlighted with red color. (TIF) [file pone.0042948.s001.tif]

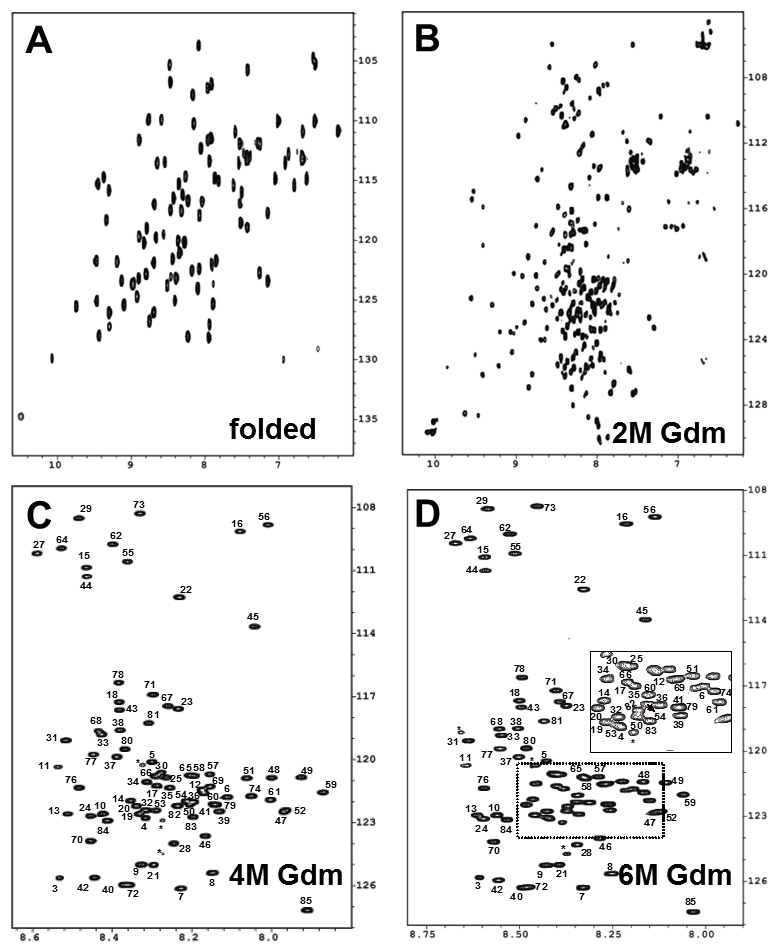

Supplement: Figure S2 — Sensitivity-enhanced 2D [15N-1H]-HSQC of [Ca2+]2-M-crystallin in the presence of 0 (A), 2 (B), 4 (C) and 6 M (D) GdmCl (pH 5.5 and temperature 298 K). These spectra were recorded on a Bruker Avance 800 MHz spectrometer with 128 and 1024 points along t1 and t2 dimensions, respectively. Individual peak assignments are shown by the corresponding single-letter code of the amino acid residue and its sequence number along the primary sequence. (TIF) [file pone.0042948.s002.tif]

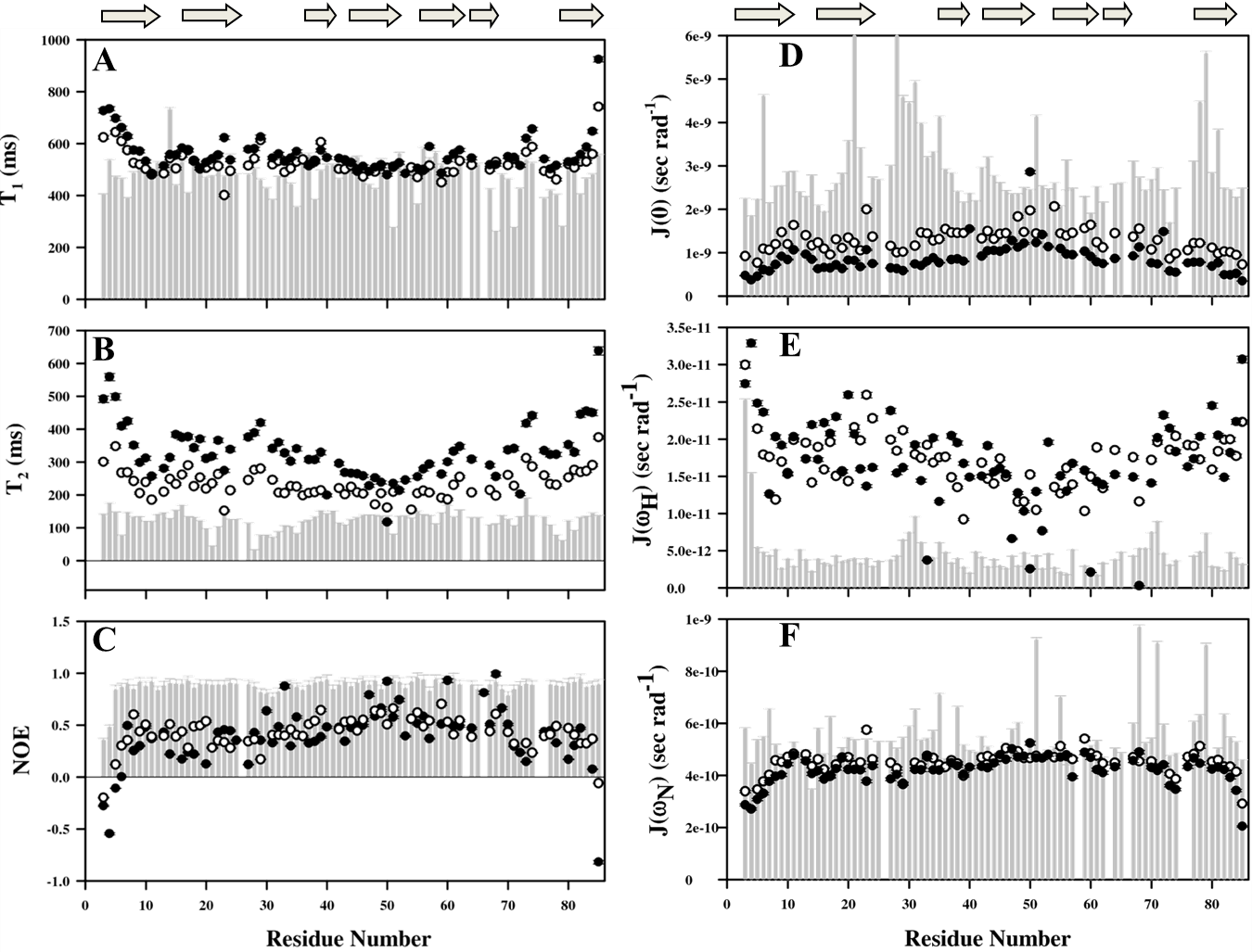

Supplement: Figure S5 — 15N relaxation parameters and reduced spectral densities versus residue number. (A) Longitudinal relaxation times (T1). (B) Transverse relaxation times (T2). (C) [1H-15N] NOE enhancements derived from Isat/Ieq ratio, where Isat and Ieq are the intensities of peaks in the 2D spectra recorded with and without proton saturation, respectively. Error bars in the T1 and T2 data denote curve-fitting uncertainties: errors in the [1H-15N] NOEs are estimated from the signal/noise ratio of the spectra. Spectral densities are shown in (D) J(0), (E) J(ωH), and (F) J(ωN), as a function of protein sequence number. Here, horizontal bars represent the data for the protein with 0 M GdmCl, filled and open circles correspond to the protein taken in 4 and 6 M GdmCl concentrations, respectively. Secondary structural elements of folded protein are depicted on top of the panels. (TIF) [file pone.0042948.s005.tif]

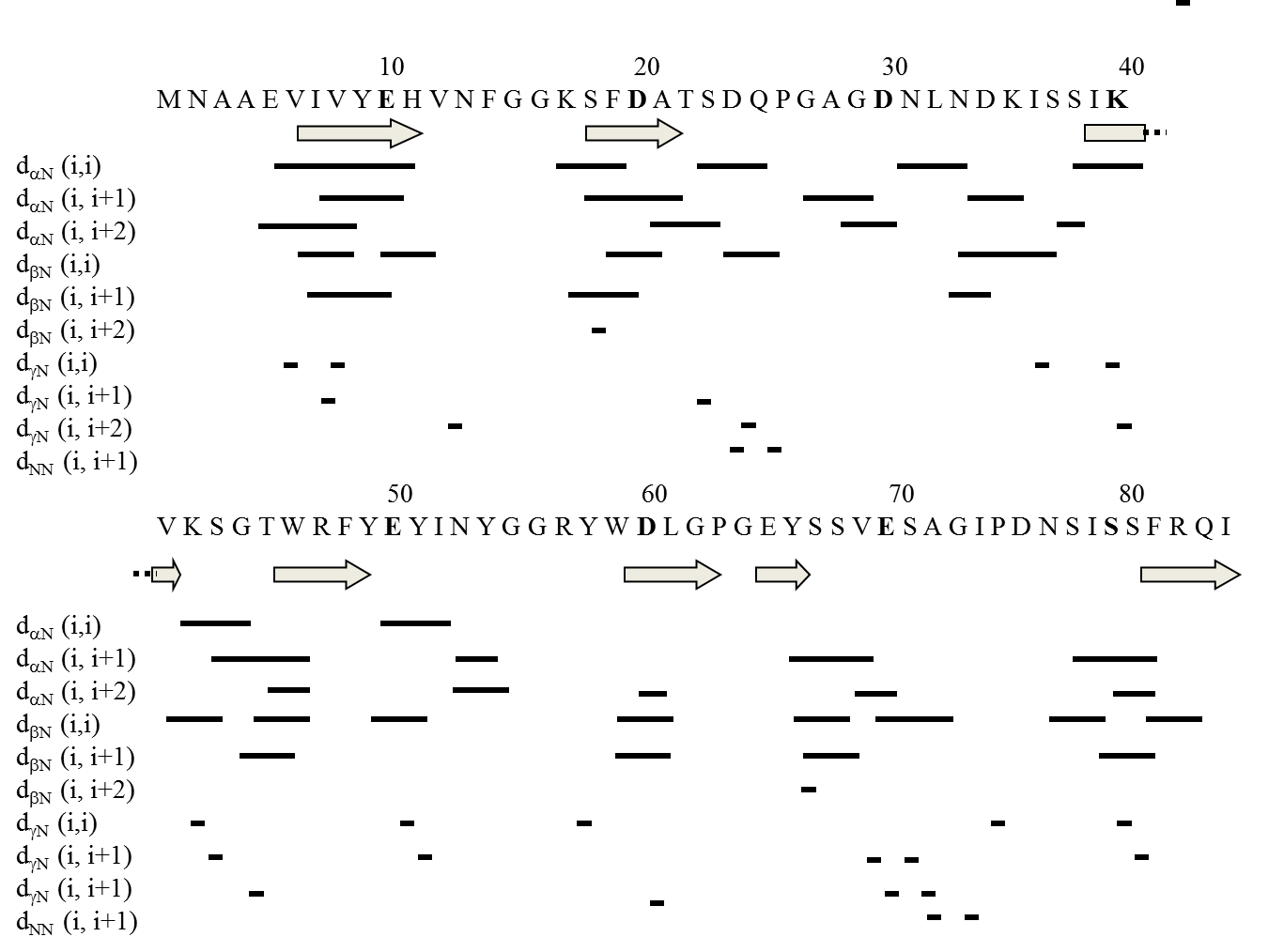

Supplement: Figure S6 — NOE connectivities obtained from 15N-edited 3D NOESY-HSQC of M-crystallin taken in 6 M GdmCl (pH = 5.5; temperature = 25 °C). The native secondary structure elements are depicted on top of the panel with arrows. Folded protein has 7 stretches of β-strands. (TIF) [file pone.0042948.s006.tif]
